# A Multicenter Retrospective Study on the Prognosis of Stage III Unresectable Mutant Non-Small Cell Lung Cancer With Tyrosine Kinase Inhibitors Therapy

**DOI:** 10.3389/fonc.2021.692703

**Published:** 2021-07-12

**Authors:** Ranpu Wu, Shaorong Yu, Jinjun Ye, Yimin Wang, Zhiting Zhao, Hongbing Liu, Yong Song

**Affiliations:** ^1^ Department of Respiratory and Critical Care Medicine, Jinling Hospital, School of Medicine, Southeast University, Nanjing, China; ^2^ Department of Medical Oncology, Affiliated Cancer Hospital of Nanjing Medical University & Jiangsu Cancer Hospital & Jiangsu Institute of Cancer Research, Nanjing, China; ^3^ Department of Respiratory and Critical Care Medicine, Affiliated Jinling Hospital, Medical School of Nanjing Medical University, Nanjing, China

**Keywords:** NSCLC, stage III unresectable, EGFR, TKI, surgery

## Abstract

**Background:**

For unresectable stage III non-small cell lung cancer (NSCLC), concurrent chemoradiotherapy is nowadays the standard treatment. Patients with advanced NSCLC harboring driver-gene mutations benefit from Tyrosine Kinase Inhibitors (TKIs) Therapy. In a real-world setting, there is room for exploring the benefit of TKIs in stage III unresectable NSCLC patients with mutation.

**Methods:**

A total of 81 patients from the Jinling Hospital and the Jiangsu Cancer Hospital with stage III unresectable mutant NSCLC applied targeted therapy were enrolled in this retrospective study. Patients with first-line application of TKIs were followed up to gain the situation of surgery qualifications, progression-free survival and overall survival, so as to evaluate the survival prognosis, then whether patients benefit and what kind of patients benefit most from TKI monotherapy treatment or its combination are explored.

**Results:**

The median progression-free survival of involved 81 patients was 13.87 months (95% confidence interval (CI): 11.66–16.08), and the median survival was 41.47 months (95%CI: 20.11–62.83). The 5-year survival rates were 91.0, 80.3, 56.1, 45.5, and 32.5%, respectively. After first-line TKI therapy, seven patients (8.6%) were reevaluated as eligible for surgery and proceeded to surgery. Although no characteristics were found to be statistical prognostic, younger female non-smokers still tended to have a better prognosis with longer progression free survival and overall survival.

**Conclusions:**

TKIs are a viable option for mutant stage III unresectable NSCLC patients who have achieved good clinical benefit from TKI. Patients who cannot tolerate chemoradiotherapy, especially those with driver gene mutations, can choose targeted therapy for first-line treatment.

## Introduction

Lung cancer is currently one of the most malignant tumors, while five-year survival at all stages was about 19%. Once metastases are diagnosed, the rate directly declines to 5% (1). Non-small cell lung cancer accounts for 80–85% of lung carcinoma (2). The recognized mutant genes in NSCLC include EGFR, ALK, ROS, HER2, etcetera. As for EGFR mutation, the most typical types are exon 19 deletion and exon 21 mutation (3–5). Epidermal growth factor receptor tyrosine kinase inhibitors (EGFR-TKIs) are commonly used in advanced NSCLC with EGFR mutation (6). While the 5-year overall survival rate of standard concurrent chemoradiotherapy wandered around 20% (7), the long-term prognosis were 15–35% for unresectable or inoperable stage IIIA and 5–10% for stage IIIB (8). In unresectable ones, further studies are still needed that whether patients with driver gene mutation can benefit from first-line treatment with EGFR TKIs.

Previous studies have explored multiple treatments for stages III/IV advanced lung cancer. The Pacific study (9) showed that among patients with stage III unresectable NSCLC, the combination of standard concurrent chemoradiotherapy and durvalumab significantly prolonged overall survival compared with placebo (hazard ratio: 0.68; P = 0.0025) with similar safety as placebo. Meanwhile, previous studies have demonstrated no further benefit in survival prolongation with combination therapies based on CCRT or afatinib, like pembrolizumab (10), or cetuximab (11). Also, increasing the radiation dose to 74 Gy (high dose) paradoxically decreased survival compared with CCRT (12).

In this study, we collected eligible patients from two centers and included patients receiving first-line treatment with targeted drugs, then analyzed the clinical benefits of TKI therapy in these patients, especially the prognosis, and possible factors affecting survival outcomes.

We present the following article in accordance with the original article reporting checklist.

## Method

### Patients

In this study, the records of patients with NSCLC (including adenocarcinoma versus squamous) diagnosed by puncture and pathology admitted to the Jinling Hospital and the Jiangsu Cancer Hospital from January 1, 2005 to December 31, 2020 were retrieved. After sieve dereplication, stage III patients were selected according to TNM staging criteria of the UICC/AJCC 8th Edition (13). The driver gene detection results were then checked to exclude patients without mutations or untested, and to reserve those with positive driver genes (EGFR, ALK, ROS, HER2). Finally, we included patients with TKI therapy or TKI combination with chemotherapy as the first-line treatment. The study was approved by the Ethical Review Committee of the Affiliated Jinling Hospital (DBNJ20219).

### Data Collection

We retrieved the medical records of the included 81 patients to obtain basic information such as age, gender, stage, driver gene, and mutation types. The specific medical records were then reviewed to obtain information on first-line treatment options, progression-free survival, second-line treatment, and whether or not surgery was performed, etc. Finally, patients were followed up for missing prognostic information such as overall survival, complication, and quality of life.

### Statistical Analysis

Survival curves were plotted with Kaplan–Meier analysis, obtaining the median survival and the median progression-free survival, with 95% confidence intervals for both OS and PFS. Cox regression was used to explore the effect of factors such as gender, age, stage, genotype, smoking, chemotherapy combination and surgery on overall survival. All statistical analyses were calculated by SPSS statistics, and all statistical tests were all two-sided, and P values <0.05 would be considered statistically significant, with 95% confidence intervals.

## Result

**Figure 1:**
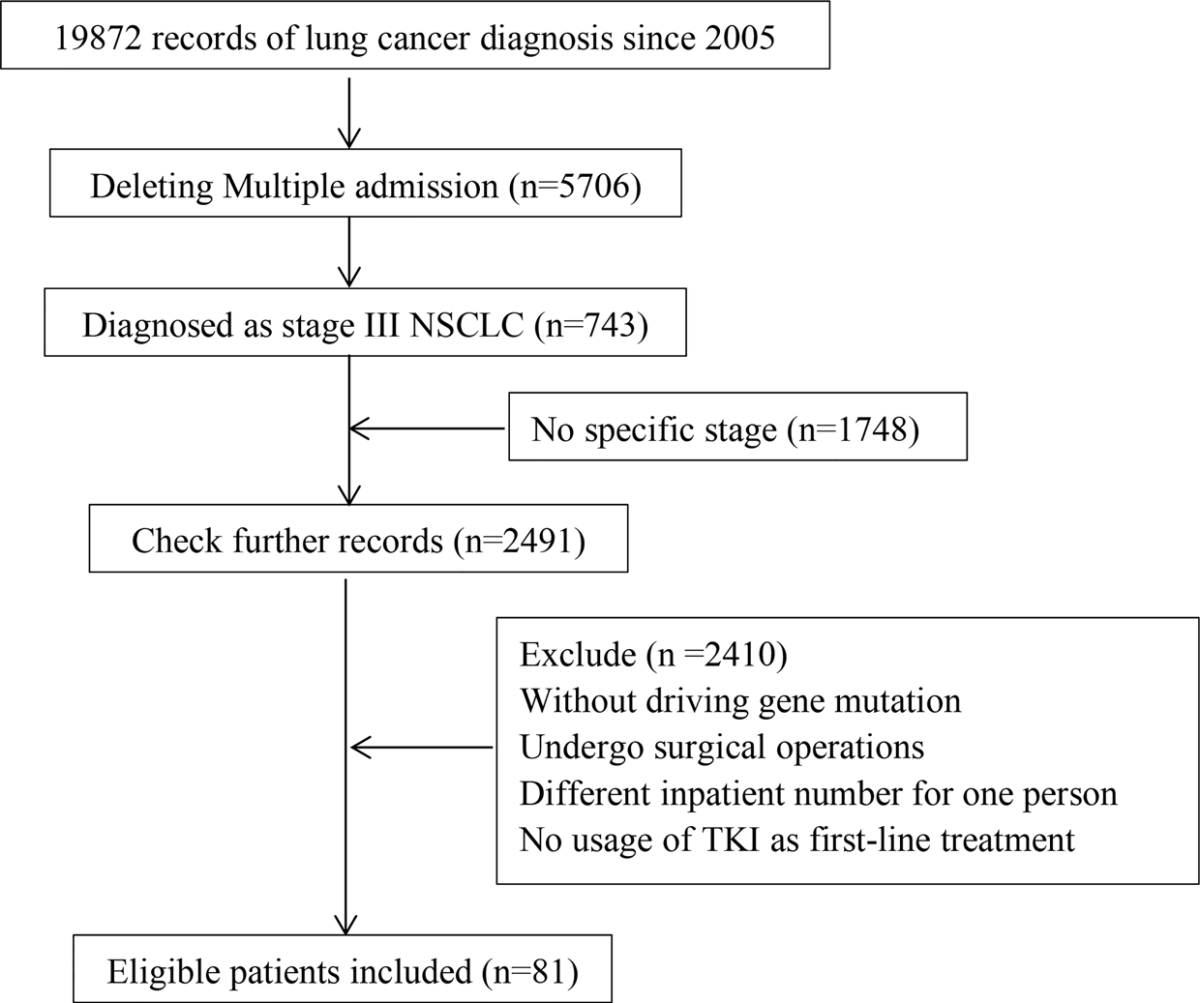
The flowchart of this study. Including collection, screening, follow-up of in volved patients.

A total of 5,706 patients who had been admitted to our hospital were screened out from 19,872 admission records, and 81 were finally included. For patients who had already died, the specific overall survival was calculated, and for those who survived, the survival was calculated with January 31, 2021 as the end point of follow-up. As for patients who recently lost to follow-up, survival was defined as the time from the last follow-up. The flowchart of this study is shown in **Figure 1**.

### Patient Characteristics

We collected the characteristics of 81 patients, as presented in **Table 1**. In this study, we included 42 (51.9%) elderly patients (≥65 years old) and 39 (48.1%) middle-aged patients. Among them, there were 20 cases of stage III A NSCLC (24, 7%), 45 cases of stage III B (55.6%) and nine cases of stage III C(11.1%); there were 35 males (43.2%) and 46 females (56.8%); Smoking, as an important factor, was also included in the study, with 11 smokers (13.6%), 57 non-smokers (70.4%) and 13 ex-smokers (16.0%).

**Table 1:** Characteristics of involved 81 patients.

Characteristic	Number (%)	Characteristic	Number (%)
**Overall**	81		
**Gender**		**Mutant gene**	
** Male**	35 (43.2)	**EGFR**	69 (85.2)
** female**	46 (56.8)	**ALK**	9 (11.1)
		**ROS**	1 (1.2)
		**HER2**	2 (2.5)
**Age**		**First-line treatment**	
** Middle-age**	39 (48.1)	**TKI**	63 (77.8)
** Elderly**	42 (51.9)	**TKI + chemotherapy**	9 (11.1)
		**TKI + radiotherapy**	6 (7.4)
		**TKI + immunotherapy**	2 (2.5)
		**TKI+ chemoradiotherapy**	1 (1.2)
**Smoke**	11 (13.6)	**Generation of TKI**	75 (92.6)
** Smoker**	57 (70.4)	**First-generation**	6 (7.4)
** Non-smoker**	13 (16.0)	**Second-generation**	
** Ex-smoker**			
**Stage**		**Surgery**	
** Ⅲ A**	20 (24,7)	**Surgery**	7 (8.6)
** Ⅲ B**	45 (55.6)	**No surgery & unknown**	68 (89.1)
** Ⅲ C**	9 (11.1)		
** Missing**	7 (8.6)		

Basic information of patients was obtained by retrieving medical records like gender, age, smoking, stage, mutation gene, first-line treatment, generations of TKIs, or surgery.

Meanwhile, all 81 included patients had driver gene mutations, including 69 EGFR mutation (85.2%), 28 classical exon 19 deletions (34.6%), 17 exon 21 mutations (21%). In addition, there were nine ALK mutations (11.1%), one ROS (1.2%), and two HER2 mutation (2.5%).

In this study, a total of 63 patients (77.8%) chose TKI monotherapy as first-line treatment, while 18 patients were treated with TKI along with other therapies combined, including immunotherapy, chemotherapy or radiotherapy. Among them, nine (11.1%) patients received TKI along with chemotherapy, including pemetrexed, pemetrexed plus cisplatin. Six patients (7.4%) received radiotherapy on the basis of TKIs, and one patient underwent concurrent chemoradiotherapy. Immunotherapy involved only two patients, one in combination with bevacizumab, paclitaxel and the other in combination with bevacizumab alone.

Among the 81 patients, 75 (92.6%) chose first-generation TKIs as first-line treatment, with 51 (63.0%) receiving gefitinib, ten (12.3%) receiving erlotinib, and five (6.2%) receiving icotinib. Meanwhile, five patients (6.2%) received afatinib, one received ensartinib, two received second-generation TKIs, and no patients received third-generation in our study.

In second-line treatment, 17 patients continued with targeted therapy or its combination with other therapies, 20 switched to chemotherapy, and four patients were treated with combination of both. Among these 17 patients, three patients continued with first-generation TKIs, four with second-generation ones and ten with third-generation like osimertinib.

### Survival and Prognosis

We followed up 81 patients and obtained their prognostic survival. Among them, 57 patients (70.4%) experienced disease progression, whereas 24 patients (29.6%) were lost to follow-up or did not yet progress. By the last follow-up, 31 patients (38.3%) had died and the remaining 50 patients (61.7%) had not yet reached the endpoint of death.

**Figure 2:**
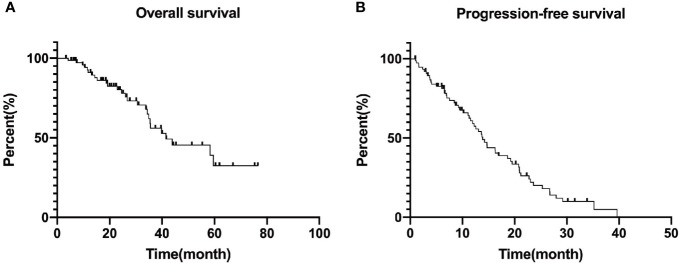
Overall survival **(A)** and median progression free survival **(B)** curves of included patients. Progression free survival: 13.87 (95%CI: 11.66–16.08) months and Overall survival: 41.47 (95%CI: 20.11–62.83) months. The survival rates for 5 years are 91.0, 80.3, 56.1, 45.5, and 32.5%, respectively.

We calculated the overall survival on January 31, 2021 as the follow-up endpoint, and plotted progression free survival and overall survival curves, as shown in **Figure 2**. The median progression-free survival of all patients was 13.87 months (95%CI: 11.66–16.08) as well as the median overall survival was 41.47 months (95%CI: 20.11–62.83). The survival rates for 5 years were 91.0, 80.3, 56.1, 45.5 and 32.5%, respectively.

At the follow-up end point, we finally found that 23 patients (28.4%) had tumor progression and metastasis development to stage IV, with eight patients presenting with brain metastases, seven patients with bone metastases, five patients with pleural metastases or malignant pleural fluid, five patients with both lungs, one patient with liver, and one patient with abdominal cavity.

### Influencing Factors of Survival

In this study, we included stage III unresectable NSCLC patients from two centers and obtained basic information and survival prognosis of these patients. Although patients treated with first-line TKI or TKI combination therapy achieved superior overall survival and outcomes in this study, some patients rapidly progressed in first-line therapy, thus affecting overall survival.

Previous studies have found that age, gender, TMN stage, smoking, surgery after first-line treatment and so on may have a statistically significant impact on survival. Subsequently, we explored possible factors which may influence survival and prognosis.

To conclude, Kaplan–Meier survival curve illustrated prolonged tendency in female patients compared with male patients in both OS and PFS, and statistical difference was found in PFS: 16.30 (11.35–21.25) *vs* 12.57 (8.90–16.24), P = 0.028. However, there is no statistical difference in OS: 59.60 (95% CI: 22.68–96.52) *vs* 35.40 (95% CI: 33.46–37.35, P = 0.065.

For elderly patients, the overall survival was 34.90 (11.68–58.12) months, and as for middle-aged patients, it was 43.87 (23.30–64.44) months, p = 0.327. Similar outcome was found in PFS: 16.27 (12.62–19.92) *vs* 12.57 (9.68–15.46) months, p = 0.220. Although P values were over 0.05, youth still probably influences prognosis.

Similarly, there is a trend of longer survival among non-smokers than that among smokers or ex-smokers. Median PFS was 11.07 (0.64–21.50) months for smokers, 16.97 (10.57–23.36) months for non-smokers, 12.23 (3.40–21.05) months for ex-smokers (p = 0.07), and median OS was 35.40 months for smokers, 58.33 (30.95–85.72) months for non-smokers; 34.90 (17.02–52.79) months for ex-smokers, p = 0.558.

In our study, 69 patients (85.2%) had EGFR mutations, and we explored the difference in survival prognosis of 28 patients (34.6%) with classic 19 deletion, compared with 17 patients (21%) with 21 mutations. The results showed that the PFS of the patients with 19 deletions was 16.27 (11.61–20.93) months, 11.93 (9.07–14.79) months in 21 mutations ones and 12.57 (5.61–19.53) months in other types of patients, p = 0.803. Overall survival for patients with 19 deletions was 58.33 (37.21–79.45) months, 21 mutations: 34.37 (12.49–56.25) months, p = 0.126. Patients with 19 deletions numerically survived longer and showed better outcome than patients with 21 mutations or other types.

In the Kaplan–Meier analysis, we found that gender and smoking might be prognostic factors for PFS and OS, so we conducted Cox regression analysis to further explore. We included gender, age, TMN stage, genetic subtype, smoking, first-line treatment, surgery, and also complications in the COX regression model, and calculated and analyzed hazard ratios and their 95% confidence intervals.

According to the results of the regression analysis, unfortunately, no influence was exerted on OS and PFS by all the factors: gender (p = 0.352/0.123, HR:0.682/0.354), age (p = 0.231/0.201, HR:1.495/2.130), TMN stage (p = 0.715/0.261), genotype: (p = 0.782/0.130), whether smoking (p = 0.462/0.507), first-line treatment (p = 0.923/0.646), whether surgery was performed (p = 0.967/0.977, HR:0.977/0.949), and complication (p = 0.112/0.212, HR:1.706/0.422).

**Figure 3:**
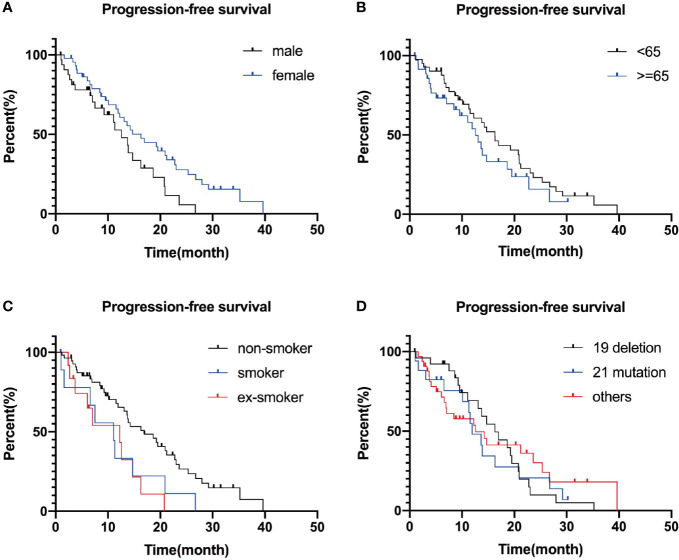
Survival curves plotted separately by gender **(A)**, age **(B)**, smoking **(C)**, and genotype group **(D)**. P-values are 0.124, 0.168, 0.929, and 0.094, successively.

**Figure 4:**
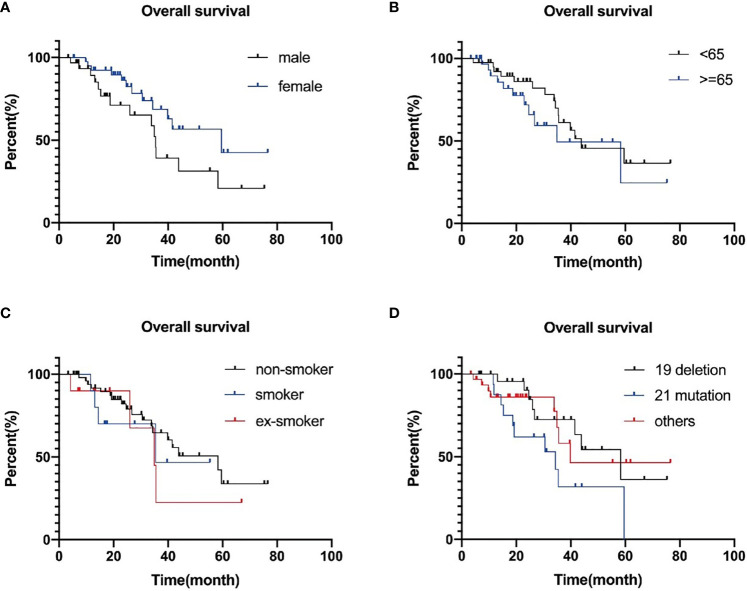
Progression-free survival curves plotted separately by gender **(A)**, age **(B)**, smoking **(C)**, and genotype group **(D)**. P-values are 0.927, 0.299, 0.126, and 0.923, successively.

To conclude, although we intended to find the TKI benefit population to guide the subsequent clinical choice, in our study, no independent factors were proven to have a prognostic impact statistically. Specific P values are listed in **Table 2**, and survival curves are plotted in **Figures 3** and **4**.

**Table 2:** The association between overall survival and progression free survival was calculated by Cox regression models.

Factors	P1 value	HR	95% CI	P2 value	HR	95% CI
**Gender**	0.352	0.682	0.305–1.525	0.123	0.354	0.095–1.323
**Age**	0.231	1.495	0.775–2.884	0.201	2.130	0.669–6.783
**Stage**	0.715			0.216		
**Genotype**	0.782			0.130		
** 19deletion**	0.829	0.921	0.436–1.946	0.811	0.826	0.171–3.963
** 21mutation**	0.655	1.225	0.504–2.977	0.170	2.991	14.304
**Smoke**	0.462			0.507		
** Smoker**	0.242	0.526	0.180–1.543	0.261	0.354	0.058–2.164
** Ex-smoker**	0.312	0.511	0.139–1.876	0.667	0.642	0.085–4.83
**Firstline**	0.923			0.646		
**Surgery**	0.967	0.977	0.334–2.856	0.963	0.949	0.105–8.580
**Complication**	0.112	1.706	0.882–3.299	0.212	0.422	0.109–1.636

P1 value, P value of overall survival; HR, hazard ratio; 95% CI, 95% confidence intervals; P2 value, P value of progression free survival. P values <0.05 were considered statistically significant, with 95% confidence intervals.

### Prognosis of Operated Patients

In 81 patients of this study, after first-line TKI therapy, seven (8.6%) patients were reevaluated as eligible for surgery and proceeded to surgery who were three males and four females. By the end of the follow-up, two patients had reached the end point of follow-up and five patients were alive. The second patient had a survival of 34.37 months. Five patients had already progressed postoperatively, including abdominal cavity, brain, multiple bone metastases and recurrence in the postoperative stump, and the progression time was 29.20, 13.73, 7.5, 6.60, and 12.23 months successively. One patient was followed up for almost three years with no evidence of progression or metastases. The specific information of the seven patients is shown in **Table 3**.

**Table 3:** Prognosis of seven operated patients.

N	Gender	Age	Stage	TMN staging	Stage after surgery	TMN Staging after surgery	Gene	First-line treatment	metastasis	Living	PFS	PFS status	OS	OS status
**1**	Female	50	IIIA	T1bN2M0	IIB	T1aN2M0	EGFR	Icotinib	Unknown	Dead	8.67	0	/	1
**2**	Female	63	IIIB	T4N2M0	IIIB	T4N2M0	EGFR	Gefitinib	Abdominal	Dead	29.20	1	34.37	1
**3**	Male	46	IIIB	T3N2M0	IIIA	T1cN2M0	EGFR	Gefitinib + AP	Brain	Living	13.73	1	22.57	0
**4**	Male	36	IIIB	T1cN3M0	/	/	EGFR	Gefitinib	Progress	Living	7.50	1	16.67	0
**5**	Male	56	IIIB	T4N2M0	IIIA	T1bN2M0	EGFR&HER2	Gefitinib + AP	Bone	Living	6.60	1	17.60	0
**6**	Female	63	IIIA	T1N2M0	/	/	EGFR	Gefitinib	No metastasis	Living	/	0	44.23	0
**7**	Female	54	IIIA	T2N2M0	/	/	EGFR	Gefitinib	Recurrence	Living	12.23	1	12.77	0

AP, Pemetrexed + cisplatin; PFS, progression-free survival, definite from the start of first-line therapy to disease progression; OS, overall survival, definite from pathological diagnosis to patient death.

Limited by the sample size and follow-up time, there was no statistically significant difference in overall survival between surgical and nonsurgical patients in this study. When considering TKIs as first-line therapy for patients with advanced NSCLC, the timing and effectiveness of surgery remain unclear.

### Complications

For the 81 patients included, 25 (30.9%) developed complications in the first-line treatment. The major complications that occurred were hepatotoxicity and rash. Nine of these patients were detected abnormal liver enzyme indices, eight with TKI monotherapy and one with TKI combination chemotherapy. Three of them were finally forced to withdraw from first-line treatment due to continuing liver damage even with hepatoprotective treatment.

The second most serious complication was rash, which was observed in seven patients during follow-up. There were two cases of oral ulcer, one of gingival bleeding and one of thrombocytopenia and one of leukocytopenia. After apatinib administration, one patient had hypogeusia and hoarseness. Notably, among the six patients with TKI combined radiotherapy, two (33.3%) of them had inflammatory changes on chest X-rays, which were considered as radiation pneumonitis.

Due to the limited sample size, we failed to analyze and conclude the occurrence of complications may associated with the combination of chemotherapy, radiotherapy or immunotherapy.

## Discussion

This study was a multicenter, real-world retrospective study involving stage III unresectable NSCLC patients carrying mutations in EGFR/ALK/ROS genes, recruited from the Jinling Hospital and the Jiangsu Cancer Hospital in Nanjing, Jiangsu Province. Our aim was to explore the prognosis of such patients after first-line applying first/second-generation TKIs or their combined therapy like chemotherapy, immunotherapy or radiotherapy, then explore which patients could clinically benefit most.

The treatment for unresectable stage III/IV advanced NSCLC is the focus of many investigators. Currently, stage III unresectable NSCLC are treated with concurrent chemoradiotherapy (CCRT) as the standard therapy (14). The investigators explored a combination of chemoradiotherapy that involved pembrolizumab, cetuximab and increased radiation dose. However, no other treatment is superior except durvalumab.

In 2018, Durm et al. (15) reported the results of a phase II study using pembrolizumab after CRT in 93 unresectable stage III NSCLC patients. The median OS was 22.4 months, the median PFS was 17 months, and the 2-year survival rate was 61.9%. Prolonging pembrolizumab treatment may be associated with prolonged PFS and OS, but unfortunately, not all patients benefit from consolidation immunotherapy (10). Studies also found that, combining with anti-EGFR antibody cetuximab did not improve overall survival (12). When the radiotherapy dose was increased from the standard dose of 60 to 74 Gy, the median overall survival was 28.7 months and the 2-year survival rate was 58%, which was better than expected. But this therapy still did not improve overall survival and was potentially harmful. The phase III PROCLAIM study (16) also demonstrated that chemotherapy consolidation following pemetrexed cisplatin or etoposide cisplatin + radiotherapy was similarly not superior to standard chemoradiotherapy. Durvalumab in combination with a PD-L1 inhibitor after concurrent chemoradiotherapy significantly increase ORR and prolonged PFS and OS (HR: 0.68; P = 0.00251) (17–19). Other studies also demonstrated that, durvalumab was safe in patients and even similar to placebo in unresectable stage III NSCLC (20, 21).

Meanwhile, TKIs such as gefitinib and afatinib are also the standard first-line treatment options for advanced NSCLC patients with driver-gene mutations (22, 23). However, the clinical benefit of first-line administration of TKI therapy in such patients remains uncertain.

A retrospective study found that 56.6% of patients chose targeted agents as first-line therapy (4). Despite the average of acquired resistance after 9 to 14 months of EGFR-TKI therapy (24), previous studies have found that the application of targeted agents is still associated with a favorable survival benefit (25).

With the further development of targeted agents research, the emergence of second-generation and third-generation TKIs provided more options for patients and urged for clinical validation. Another real-world retrospective study (26) in 2020 analyzed 620 stage III/IV NSCLC patients with EGFR mutated. All patients had a PFS of 11.6 months and a median OS of 19.4 (17.5–21.7) months. The median PFS and 1 year survival rate for the three groups were: gefitinib 10.3 months, 69.1%; erlotinib 12.1 months, 71.6%; and afatinib 16.4 months, 78.2%. The median OS was 20.4 (17.5–27.8) months in the erlotinib arm and 17.5 (15.2–20.3) months in the gefitinib arm. Although the OS of afatinib arm was not reached in the study, it could still be concluded that afatinib had an advantage in prolonging PFS in patients. In LUX-Lung 7 (27), there was no significant difference in OS between afatinib and gefitinib, which was similar to the conclusion of our study. In our study, gefitinib was administered to 62.9% of patients. We explored the relationship between survival in patients treated with first or second generation TKIs, but found no statistical difference of PFS or OS (p = 0.903/0.799).

As for second-line TKIs, five patients were applied to afatinib, while no patient in the study received dacomitinib. Dacomitinib, a second-generation EGFR-TKI/HER2-TKI, was already approved in the United States, Japan and the European Commission for first-line monotherapy treatment in patients with EGFR mutation-positive inoperable or recurrent NSCLC, which may be a powerful new treatment option compared with gefitinib (28, 29). As a rare gene mutation, HER2 mutation was found in two patients included in our study, but both of them chose afatinib as first-line therapy. Although no patient received third-generation TKI monotherapy for first-line treatment in this study, the use of third-generation TKIs was clinically increasing. Unfortunately, a phase III clinical trial of a third-generation novel agent ASP8273 versus erlotinib or gefitinib in patients with advanced stage IIIB/IV NSCLC had to be discontinued due to excessive toxic side effects (30).

Meanwhile, other studies also found that in advanced NSCLC, different EGFR mutation types had different response rates to second-generation EGFR TKI like afatinib, in particular del 19 (31). But no difference of OS in first-line generation TKI was found between del 19 and L858R mutation (3) in this study, neither was found in our study.

There was no statistically significant difference in PFS or OS between del 19 and 21 mutation (p = 0.782/0.130).

A retrospective study compared the efficacy of TKIs monotherapy with CCRT in patients with stage IIIB lung adenocarcinoma with EFGR mutation. Although the 5-year OS rates in the TKI group compare with the CCRT group increased at a numerical level (30 and 26%), there was no statistically difference between the two groups (32). Considering that our study was just a single-arm, retrospective study and lacked a feasible chemotherapy control arm, we may not further conclude a survival advantage of EGFR TKIs monotherapy or combination therapy over CCRT. Since the poor response to monotherapy, many researchers turned to explore the possibility of combining TKI with other treatments. Whether the combination of chemotherapy and radiotherapy obtain more clinical benefits than single drug is a concern of many researchers. A multicenter phase II clinical trial compared the median OS of concurrent radiotherapy plus erlotinib with chemoradiotherapy. It was found that erlotinib group improved PFS, while patient tolerance of both groups was similar. Along with radiotherapy, TKI therapy showed better clinical benefits than chemotherapy in prolonging PFS. As for radiotherapy, radiation pneumonitis is one of the most serious complications affecting patient survival. Previous studies found that the incidence of radiation pneumonitis in patients with radiotherapy combined with first-generation TKI is as high as 40%, and the incidence of those over grade 3 is 20% (33). Among the six patients who were treated with radiotherapy along with TKI in our study, two patients showed inflammatory reactions on radiologic level, but none of them were severe but mild. RECEL study (34) found that erlotinib combined with thoracic radiotherapy significantly improved the median PFS compared with chemoradiotherapy, which suggested the value of EGFR-TKI with concurrent radiotherapy. In another retrospective study (35), among 45 unresectable stage III NSCLC patients receiving radiotherapy with EGFR-TKI, 17 patients (37.7%) suffered radiation pneumonitis, but achieved satisfactory PFS and OS: 27.9 (95% CI: 18.7–37.2) and 49.7 (95% CI: 27.7–71.8) months. A new phase II clinical trial (NCT0463659) is exploring the safety and efficacy of almonertinib and concurrent thoracic radiotherapy in patients with unresectable stage III EGFR-mutated NSCLC (36). If the patient is well tolerated, a combination of targeted drugs and radiotherapy may be chosen as first-line treatment. Further study may provide more objective basis for targeted therapy combined with radiotherapy in the treatment of advanced lung cancer.

Smoking status is a significant predictor of response and survival after underdoing EGFR TKIs treatment (37, 38). Previous studies have found that cigarette smoking dosage over 30 pack-years was an independent negative predictive factor and meanwhile smoking cessation combined with anti-EGFR treatment like erlotinib seems to be more effective in lung adenocarcinoma with EGFR mutation (39, 40). In two randomized trials comparing gefitinib (41) or erlotinib with placebo, non-smokers had a significant survival benefit. Our study showed similar outcomes to previous researches (42) with a prolongation of median non-smoking PFS with OS compared to those who smoked or quit, although they were not statistically different (p = 0.462/0.507), indicating that TKI therapy had better clinical benefits for non-smokers with positive driver gene. We also observed the prolongation of progression free survival and overall survival in female and middle-aged patients, but there was no statistical difference in the end.

Surgery has been widely used as radical treatment for tumor patients of resectable diseases. Preoperative and postoperative adjuvant chemotherapy seemed to have significant survival benefit (43, 44). Among stage III lung cancer patients, only some of them have access to surgery. Previous studies such as Yamamoto’s (45) and Mazzoni’s (46), reported that some patients regain the opportunity to undergo surgery after first-line treatment and finally excised. A multicenter study (47) showed that 14.8% of stage III patients underwent surgery. In previous studies, there was no significant difference in PFS between the surgery and non-surgery groups (HR = 0.91, 95% CI: 0.73–1.13) (48). In our study, seven (8.6%) patients ultimately underwent surgery. However, limited by the sample size, there was no benefit in PFS or OS between surgery patients and others (p = 0.967/0.977).

This study was a two-center retrospective study with a limited number of included patients, large interpatient heterogeneity and a proportion of patients lost to follow-up. Our study supported that the application of TKIs in stage III unresectable NSCLC patients with positive driver gene may achieve a good clinical benefit and is a considerable option. For patients who cannot bear chemoradiotherapy, especially those who have never smoked, if EGFR/ALK mutations occur, anti-EGFR/ALK therapy is considered as first-line treatment. However, further larger studies are still needed to validate this conclusion and explore the optimal treatment regimen for stage III unresectable mutant patients.

## Data Availability Statement

The raw data supporting the conclusions of this article will be made available by the authors, without undue reservation.

## Ethics Statement

The studies involving human participants were reviewed and approved by Ethical Review Committee of the Affiliated Jinling Hospital (DBNJ20219). Written informed consent for participation was not required for this study in accordance with the national legislation and the institutional requirements.

## Author Contributions

(I) Conception and design: RW. (II) Administrative support: None. (III) Provision of study materials or patients: SY and JY. (IV) Collection and assembly of data: RW. (V) Data analysis and interpretation: None. All authors contributed to the article and approved the submitted version.

## Funding

This work was supported by the National Nature Science Foundation Of China (81772500); Natural Science Foundation of Jiangsu Province (BK20180139); Department of science and technology of Jiangsu Province (BE2019718, BE2019719).

## Conflict of Interest

The authors declare that the research was conducted in the absence of any commercial or financial relationships that could be construed as a potential conflict of interest.
